# The landscape of rare mitochondrial DNA variants in sudden cardiac death: A potential role for ATP synthase

**DOI:** 10.1016/j.heliyon.2024.e41592

**Published:** 2024-12-31

**Authors:** Elena Luppi, Monica De Luise, Carla Bini, Guido Pelletti, Gaia Tioli, Ivana Kurelac, Luisa Iommarini, Susi Pelotti, Giuseppe Gasparre

**Affiliations:** a Unit of Medical Genetics, Department of Medical and Surgical Sciences, University of Bologna, Bologna, Italy; b Unit of Legal Medicine, Department of Medical and Surgical Sciences, University of Bologna, Bologna, Italy; c Department of Pharmacy and Biotechnology, University of Bologna, Bologna, Italy

**Keywords:** Sudden cardiac death, Mitochondrial genome, M chromosome, Mitochondrial haplogroups, SNVs, Heart disease

## Abstract

Sudden cardiac death (SCD) is a major health concern, which can be the sign of a latent mitochondrial disease. However, mitochondrial DNA (mtDNA) contribution is largely unexplored in SCD at population level. Recently, mtDNA variants have been associated with congenital cardiopathy and higher risk of ischemic heart disease, suggesting them as potential risk factors also in SCD. Therefore, we aimed to define the mtDNA mutational landscape in such phenotype, by sequencing the whole blood mtDNA genome in a pilot cohort of 28 unrelated subjects. Coding variants were prioritized according to their population and haplogroup frequency. Out of 28 patients, 36% were diagnosed with coronary artery disease, 39% with structural defects and 25% with unspecified cardiac disease. The overall frequency of macro-haplogroups followed the distribution in the European population. No known or novel mtDNA pathogenic variants were found. Two rRNA and 8 missense variants were rarer than polymorphisms as they had a frequency lower than 1% in population databases. 5/8 missense variants clustered in ATP synthase genes and 4/8 missense variants were previously detected in patients with suspected mitochondriopathy. We concluded that primary mitochondrial disease is not a major cause of SCD, but rare mtDNA variants may occur (35.7% in our cohort vs 0.65% in the population; p < 0.01), potentially modifying the risk.

## 1 Introduction

Sudden Cardiac Death (SCD) is a major health concern, accounting for 15–20% of overall mortality with population estimates ranging between 50 and 100/100.000 persons *per* year in Western countries [[1], [2], [3]]. SCD is defined as a natural unexpected event occurring within 1h of the witnessed onset of new or worsening symptoms, or, if unwitnessed, within 24h of last being seen alive, for which extracardiac conditions have been excluded [[2], [3], [4]]. Upon autopsy, the cardiac phenotypes, whose prevalence varies according to age, can be grouped into three major categories encompassing coronary artery disease, non-ischaemic structural heart diseases (i. e. cardiomyopathy, myocarditis, congenital heart disease, valvular disease), and unexplained cardiac death deemed of arrhythmic origin [3,4]. According to aetiology, SCD may be the result of common acquired conditions, usually multifactorial, or the consequence of genetic diseases such as primary mitochondriopathies. The latter can either manifest as multisystem syndromes or involve only one or few organs, in some cases showing SCD as the sign of a latent disorder [5,6]. A substantial fraction of mitochondriopathies is due to mitochondrial DNA (mtDNA) alterations [7], which have been associated with susceptibility to commonly acquired conditions including congenital cardiopathy and ischemic heart disease [8,9] (IHD). Furthermore haplogroup variation has been linked to differences in oxygen consumption rate, suggesting an influence of mtDNA variability on cardiovascular risk [6,10]. MtDNA variants may therefore constitute a potential risk factor also in SCD, albeit mtDNA variation contribution is largely unexplored in SCD at population level. Sporadic studies describe few rare mtDNA variants and attempt to establish the predisposing role of specific sub-haplogroups, such as for instance the H1 sub-haplogroup in a Finnish SCD cohort [11].

An additional issue concerns the interpretation of low penetrant mtDNA variants that may promote a disease state when combined with additional triggers, among which other mtDNA variants or metabolic stressors as acquired factors such as, for instance, aminoglycoside exposure [[12], [13], [14]]. This is even more so relevant in cases where the haplogroup background modulates penetrance of disease-causing mtDNA variants, such as in Leber's Hereditary Optic Neuropathy [15] (LHON). To further complicate this scenario, peculiar combinations of individually non-pathogenic missense mtDNA variants were recently described to cause low penetrance LHON [16].

In this study, we defined the variation landscape in the entire germline mtDNA genome in a pilot cohort of subjects deceased of SCD, aiming to expand the knowledge of mitochondrial contribution to such disease and to identify possible causative alterations for latent mitochondrial disease, low penetrant single nucleotide variants (SNVs) or susceptibility haplogroups that may modify SCD risk.

## 2 Materials and methods

### 2.1 Study cohort

The study population included unrelated subjects deceased of out-of-hospital SCD who were referred to the Unit of Legal Medicine in Bologna (Italy) between 2018 and 2022. SCD was diagnosed following a judicial autopsy, requested by the Public Prosecutor to assess the possibility of a crime. Ancillary tests such as histological, toxicological and genetic analyses were also performed, following the protocol of the multidisciplinary network of Emilia Romagna of juvenile SCD [17].

Extracardiac causes of death were excluded by a full autopsy performed according to a shader forensic methodology [18]. The cardio-pathological analysis was performed following the 2017 guidelines of the Association for European Cardiovascular Pathology by a forensic pathologist and expert cardio-pathologist [2]. During the autopsy, biological samples including peripheral blood were collected and stored at −20°C. A general toxicological screening and quantification for alcohol, illicit drugs, and medicinal drugs were performed. Subjects who had a diagnosis of illicit drug abuse or a monogenic cardiac disease, as defined by pathogenic or likely pathogenic variants (according to Richards S. et al. [19]) in nuclear genes causing inherited cardiomyopathies or cardiac arrhythmias, were excluded to avoid selection bias and control possibly confounding factors that may complicate the biological interpretation of mtDNA variation contribution to SCD in the general population.

Ultimately, 28 SCD cases with either unavailable heart disease nuclear gene testing (1/28 due to low quality genomic DNA) or inconclusive results were included in this pilot study. Blood samples stored during diagnostic investigations were retrieved for mtDNA sequencing. The following data were retrospectively collected for each case in a pseudonymized database: age, sex, cardiac phenotype, geographical area origin (Asian, European, Latin American) and activity immediately before death (rest, moderate activity, high activity, not available).

### 2.2 Mitochondrial DNA sequencing analysis

Whole genomic blood DNA was extracted using the QIAamp®️ DNA Mini and Blood Mini kits (Qiagen, Hilden, DE) following the protocol “DNA Purification from Blood or Body Fluids”. Quality and quantity of extracted DNA were determined on a QuantStudio 5 Real-Time PCR for Human Identification (Thermofisher Scientific, Waltham, MA, USA) using the Quantifiler™️ Trio DNA Quantification Kit (Applied Biosystems, Foster City, CA, USA) according to the manufacturer's recommended protocol. Whole mtDNA Sanger sequencing was adapted from Girolimetti G et al. [20]. Briefly, 2.5–5ng of genomic DNA per PCR reaction were amplified with a set of 46 overlapping primer pairs covering the entire 16.5-kb mtDNA, using 2μl of 0.8μM solution of each primer pair in a final volume reaction of 10μl. Electropherograms were aligned to the revised Cambridge Reference Sequence (rCRS) of the human mtDNA (Ref.Seq. NC_012920.1) and analyzed with SeqScape version 2.5 software (Applied Biosystems).

### 2.3 MtDNA variant annotation and haplogroup prediction

FASTA files generated by SeqScape were input in Mitomaster [21] (https://www.mitomap.org/ accessed on January 24, 2024) for mtDNA variants annotation against the rCRS and haplogroup prediction. Sequences were submitted to the public database GenBank and their identifiers are reported in Supplemental Material 1. Haplogroup prediction was conducted according to PhyloTree Build 17 [22]. To avoid sequencing and annotation errors due to specimen cross-contamination, the mtDNA phylogeny approach was applied and mitochondrial haplogroups were assigned on the basis of homoplasmic haplogroup-defining variants [23]. To compare our cohort's halogroup distribution to a reference population, type II error was quantified. As calculated by using Kane SP. Post. ClinCalc (https://clincalc.com/stats/power.aspx. Updated June 23, 2024), to detect a 1.5–2 fold difference in haplogroup distribution when one study group was compared to the reference population, a sample size of 28, as in our case, can preserve a statistical power of at least 80% (type II error: 20%).

### 2.4 MtDNA variant prioritization

MtDNA variants were prioritized according to the criteria described in the ACMG/AMP specifications for mtDNA variants interpretation [24]. MitoTIP [25] and APOGEE 2 [26] (https://mitimpact.css-mendel.it accessed on March 23, 2024) were used for *in silico* prediction of the potential functional impact of tRNA and missense variants, respectively. Clinvar (https://www.ncbi.nlm.nih.gov/clinvar/accessed on March 23, 2024) and Clingen (https://clinicalgenome.org/accessed on March 23, 2024) databases were reviewed for variant mitochondrial disease reports and rating.

Given data source availability, tRNA, rRNA and missense variants were prioritized over non-coding and synonymous variants. According to allele frequency, two groups were retrieved: 1. rarer variants with frequency in sample-specific haplogroup branch and population databases <1%; 2. variants with frequency in sample-specific haplogroup branch <1% but commonly registered (>50%) in other haplogroups, as inferred from Mitomap GeneBank mtDNA sequences. Prioritized coding mtDNA variants were confirmed using a second PCR reaction.

Assuming an equal susceptibility to variations of all mtDNA regions, the mitochondrial gene occupancy of SNVs was calculated as the ratio of prioritized SNV frequency, counted as single values per gene by the total number of the prioritized variants, over the gene length percentage in the total mtDNA.

In order to infer a mitochondrial hotspot for rare variants in our cohort, their cumulative frequency per mitochondrial complex was compared to the frequency sum of all SNVs detected in gnomAD v3.1 (https://gnomad.broadinstitute.org/ accessed on March 23, 2024) with a rate ≤ than the highest gnomAD v3.1 frequency found in our cohort for a rare SNV. In order not to underestimate the SNV rate in gnomAD database, only the homoplasmic count was considered.

### 2.5 Amino acid change mapping and protein stability analysis

Amino acid substitutions were mapped on their respective protein structures by using UCSF ChimeraX software version 1.7.1 [27]. Human crystal structures of respiratory complex I (CI), IV (CIV) and V (CV) were downloaded from RCSB—The Protein Data Bank (http://www.rcsb.org/ accessed on March 24, 2024) using the “Fetch by ID” function (CI—5XTD, CIV—5Z62, CV—8H9S). The unfolding Gibbs free energy change (ΔΔG) was computed by PSP-GNM for prioritized variants to evaluate the effects of single amino acid substitutions on protein stability [28]. A positive ΔΔG indicates greater thermodynamic stability, while a negative ΔΔG indicates reduced stability. We appointed the variants as affecting stability when their ΔΔG exceeded the Root Mean Square Error (RMSE).

### 2.6 Statistical analysis

Statistical analyses were performed using GraphPad Prism v.8 (GraphPad Software Inc., San Diego, CA, USA). Clinical and demographic data were summarized using descriptive statistics. Frequencies and percentages were used for qualitative variable, while mean, standard error, minimum and maximum values (range) for quantitative variables. To compare categorical variables contingency tables were used and Chi square test (for frequencies ≥5) or Fisher's exact test (for frequencies <5) were performed. Statistical significance was assumed for p-values <0.05.

## 3 Results

### 3.1 Demographic and phenotypic characteristics of the study cohort

To define the mtDNA variation in a pilot Italian cohort of patients deceased of unexpected cardiac death, 28 subjects in whom forensic analysis excluded non-cardiac causes of SCD were included in this study. The cohort demographic data are summarized in Table 1.

**Table 1:** **Demographic data and cardiac phenotype of 28 subjects deceased of SCD.** Abbreviations: std. err. = standard error.

Cardiac Phenotype	N	%	Age Mean ±std err. (years)	Age range (years)
All	28	100	43 ± 4	1–76
Nationality				
o European	24	86	44 ± 4	1–76
o Latin American	1	3	22	–
o Asian	3	11	43 ± 13	16–62
Sex				
o M	23	82	42 ± 5	1–76
o F	5	18	49 ± 6	31–65
Coronaropathy	10	36	55 ± 5	33–76
Structural heart disease:	11	39	32 ± 7	1–66
o Myocarditis	3	11	10 ± 5	1–19
o Valvulopathy	2	7	18	16–20
o Anomalous course of coronary arteries	3	11	47 ± 12	24–66
o Cardiomiopathy	3	11	47 ± 13	22–65
Unspecified heart disease	7	25	43 ± 5	23–58

The mean age of death was 43 years (±4 years std. err.) with a wide age range (1–76 years) and a higher prevalence of males (82%). Upon autopsy examination, the cardiac phenotypes were categorized into three groups: 1. cases suffering from coronary artery disease (36%), defining the IC; 2. subjects displaying structural heart diseases (39%), encompassing myocarditis, congenital valvular disease, anomalous course of coronary arteries and cardiomyopathy; 3. a normal cardiovascular phenotype was observed in 25% individuals. The three groups were almost equally represented, and among them the age range showed a similar distribution, except for myocarditis and congenital valvular disease subgroups, which were associated with younger age, as expected by their incidence in the population. According to geographical area recording, most of the cohort was of European origin (24/28; 86%), while 3 subjects (11%) were from Asia and 1 (3%) from Latin America. Taken together, the study population proved to equally represent the 3 cardiac phenotypes of SCD, displaying a broad age range, and a prevalent European origin, which reflects the population distribution.

### 3.2 Macro-haplogroup distribution confirms the study cohort as representative of the european population

**Fig. 1:**
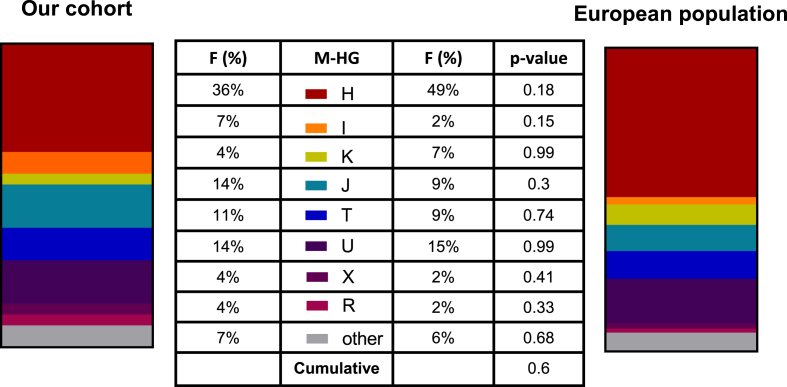
Macro-haplogroup distribution confirms the study cohort as representative of the European population. Macro-haplogroup (M-HG) distribution in our cohort (left; 28 patients) was compared to the European population (right; 1443 people calculated as the sum of mitochondrial DNA samples tested in European countries from ref. 29). Since, in the populations thereby considered, sample collection was based on geographical locations rather than their ancestry, for our comparison all subjects from our cohort were included. Different M-HG were marked with different colors according to legend (middle). Single M-HG frequencies between the two cohorts were compared using Fisher's exact test yielding no statistically significant differences (p > 0.1). Cumulative p-value at Chi square test between the two M-HG distributions is provided (p = 0.6). F: frequency.

To understand whether certain haplogroups are predisposing for SCD, we predicted the subjects’ mtDNA haplogroups at the branch level in our cohort. For the purpose of our description, all haplogroups were clustered within the corresponding macro-haplogroup, showing that H (36%), U (14%) and J (14%) were the three most represented top-level haplogroups (Fig. 1).

Our cohort, notwithstanding its small size, did not appear to differ from the general European population [29], as demonstrated by the comparison between single macro-haplogroup frequencies by Fisher's exact test (Fig. 1) and further supported by type II error quantification. In detail, according to geographical origin recordings, no statistically significant differences were observed in the top-level haplogroup distribution between the two cohorts (p = 0.6 at cumulative Chi-square test), with the haplogroup H as the most represented (Fig. 1). Furthermore, as a previous case-control study [11] found a positive association between SCD and haplogroup H1, we also investigated the frequency of the H1 sub-haplogroup in order to verify if this may be predisposing in our cohort as well. In our sample set, we were able to retrieve 2 subjects out of 28 (7%) belonging to sub-haplogroup H1 (Supplemental Material 1) and 42 out of 1443 (2%) in the European population, with no statistical differences between the two groups (p = 0.2 at Fisher's exact test).

### 3.3 Haplogroup-unrelated non-coding mtDNA variants occur in SCD

Since mtDNA SNVs may represent candidate risk factors also in SCD, we aimed to define the mtDNA variation in our pilot cohort.

Interestingly, among all variants retrieved from Sanger sequencing (Supplemental Material 2), no known pathogenic or disruptive variants typical for primary mitochondrial diseases were identified, suggesting that the latter may not be a common cause of SCD in our cohort.

According to the ACMG/AMP guidelines, a stand-alone criterion for benign classification is a variant frequency exceeding 1% in population databases or its occurrence as haplogroup-defining variant in an individual belonging to the same haplogroup. We hence focused our analysis on underrepresented SNVs, which display an allele frequency <1% for sample-specific haplogroup branch in the GenBank database, which means that SNV can be found in less than 1 to 100 mtDNA subjects’ sequences belonging to the same mtDNA background and stored in the database.

The main mtDNA non-coding region is the D-loop (nt 16024-576), in which variants affecting functionally relevant positions may impair normal mtDNA replication, negatively affecting mtDNA copy number [30]. In our cohort, mtDNA variants in such regions were scant (Supplemental Material 3); indeed, only a small subset of changes (n = 5), differing from patient-specific haplogroup background, were identified in the H-strand replication control region in 4 out of 28 subjects (14%; Supplemental Material 3). Despite the fact that a functional effect of these SNVs may not be ruled out, the pathogenicity interpretation of D-loop variants remains ambiguous, even more so when it comes to low penetrance mutations. We were therefore prompted not to include these latter into subsequent statistical associations to avoid overestimation.

### 3.4 Haplogroup-unrelated coding mtDNA variants are commonly registered in SCD

**Fig. 2:**
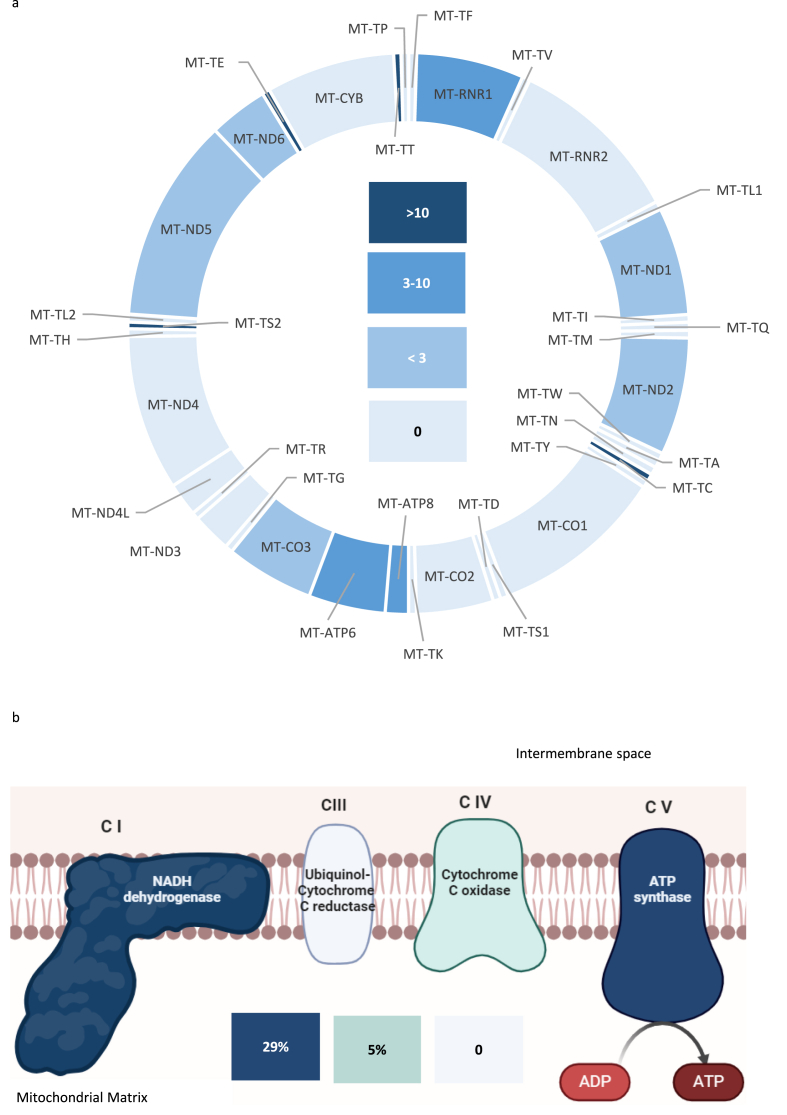
Haplogroup-unrelated coding mtDNA variants are frequent in SCD. a) Distribution of SNVs identified in 15 patients deceased of SCD according to schematic representation of coding mtDNA gene length. Only coding SNVs with non-polymorphic frequency (<1%) in patient's specific haplogroup branch were considered. Shades of blue display the ratio of SNVs frequency (number of variants per gene by the total number of the prioritized variants) over the gene length percentage in the total mtDNA. b) Distribution of missense SNVs according to the respiratory chain complexes. Only coding SNVs with non-polymorphic frequency (<1%) in patient's specific haplogroup branch were considered. Shades of blue according to the legend depict the mtDNA SNV frequency obtained as single values *per* complex over the total number of the prioritized variants. Only complexes with mitochondrial-encoded proteins are shown (adapted from Biorender.com).

Due to the potential biological impact of coding variants on protein synthesis and interpretation issues for non-coding changes, we next focused on the analysis of tRNA, rRNA, and missense variants in mitochondrial genes. Overall, 21 SNVs were identified in 15 unrelated individuals, encompassing 4 SVN in rRNA, 4 in tRNA and 13 missense variants in protein coding genes (Table 2, Table 3). All tRNA and 20/21 missense SNVs were predicted as likely benign or benign by MitoTip and APOGEE 2, respectively. As displayed in Fig. 2a, *MT-ATP6* and *MT-ATP8, MT-RNR1* and 4 tRNA genes showed nucleotide changes with a gene occupancy ratio over 3, meaning that variants in these genes occurred in our cohort at a frequency more than three times higher than expected based on the mtDNA occupancy of that gene. Similarly, albeit to a lesser extent, *MT-ND1, MT-ND2, MT-ND5, MT-ND6* and *MT-CO3* also display a trend to accumulate changes.

**Table 2:** **mtDNA SNPs with allele frequency <1% in patient-specific haplogroup branch.** All SNPs were detected in homoplasmy. AA: amino acid, MT: mitochondrial, NT: nucleotide, seqs: sequences.

Patient ID	Age (years)	Phenotype^a^	State^b^	rCRS position^c^	NT change	Locus	AA position	In silico prediction^d^	ΔΔG^e^	Patient's Haplogroup branch	Genbank Frequency % by Haplogroup branch (count/total)	Genbank common Haplogroup branch^f^	gnomAD 3.1 Frequency % (count/total)	Helix Frequency % in 195983 seqs (count)	MT Disease Report
MCI4	19	S	N/A	14053	A > G	*MT-ND5*	T573A	B	0	H7	0.000 (0/80)	H1o	0.354 (200/56425)	0.313 (613)	–
				14582	A > G	*MT-ND6*	V31A	B	−0.37		0.000 (0/80)	H4a	0.989 (558/56428)	1.243 (2436)	Yes^g^
MCI7	24	S	R	681	T > C	*MT-RNR1*	–	N/A	–	N1b	0.633 (1/158)	D5b	0.085 (48/56428)	0.068 (133)	–
MCI22	42	C	MA	5814	T > C	*MT-TC*	–	LB	–	H	0.000 (0/600)	L2b	1.377 (777/56413)	0.255 (500)	Yes^h^
MCI26	65	C	MA	3316	G > A	*MT-ND1*	A4T	B	0	T2b	0.285 (3/1053)	D2, M33c, R30a	0.457 (258/56400)	0.495 (970)	Yes^i^
MCI34	58	U	R	980	T > C	*MT-RNR1*	–	N/A	–	K2a	0.000 (0/265)	U7	0.401 (226/56421)	0.389 (763)	–
				12234	A > G	*MT-TS2*	–	B	–		0.000 (0/265)	M30c, R21	0.050 (28/56428)	0.041 (81)	–
				14687	A > G	*MT-TE*	–	B	–		0.000 (0/265)	M51a, H3k, T2a	0.792 (447/56433)	1.11 (2176)	Yes^J^
MCI35	76	C	MA	13105	A > G	*MT-ND5*	I257V	B	0	X2b	0.000 (0/271)	L0, L1, L3b, L3d, L5, R7, V2, HV5, D5c	12.75 (6556/51421)	2.491 (4882)	–
MCI9	56	U	MA	8843	T > C	*MT-ATP6*	I106T	LB	−6.5	J2a	0.465 (2/430)	A11, H45	0.512 (289/56400)	0.394 (773)	Yes^K^
MCI36	66	S	R	15930	G > A	*MT-TT*	–	B	–	T2c	0.000 (0/286)	L0d, C1c, M13b, B4d	0.860 (485/56406)	0.791 (1551)	–

a Cardiac Phenotype: C-coronaropathy, S-structural heart disease, U-unspecified heart disease.

b Physical activity: R –at rest, MA-moderate activity, HA – high activity.

c Ref.Seq. NC_012920.1.

d Apogee2 (Bianco SD et al., 2023 [26].) and MitoTip (Sonney S et al., 2017 [25].) were applied for pathogenicity prediction of missense and tRNAvariants respectively; B-benign, LB- likely benign, VUS + -variant of unknown significance with a potential pathogenic impact, N/A not available.

e ΔΔG was computed according to Mishra SK 2022 [28].

f Only haplogroups with a minimum of 10 sequences in Genbank and a variant frequency higher than 50 % were reported.

g Caporali L et al., 2018 [16].

h Scuderi C et al., 2007 [31].

i Dai Y et al., 2018 [32].

j Bruno C et al. 2003.

K Ganetzky RD et al. 2019.

**Table 3:** **Rare coding mtDNA SNVs with allele frequency <1% in population databases.** AA amino acid, MT: mitochondrial, NT: nucleotide, seqs: sequences.

Patient ID	Age (years)	Phenotype^a^	State^b^	rCRS position^c^	NT change	Homo/Heteroplasmy	Locus	AA position	In silico prediction^d^	ΔΔG^e^	Patient's Haplogroup branch	Genbank Frequency % by Haplogroup branch (count/total)	gnomAD 3.1 Frequency % (count/total)	Helix Frequency % in 195983 seqs (count)	MT Disease Report
MCI7	24	S	R	789	T > C	+/−	*MT-RNR1*	–	N/A	–	N1b	0.633 (1/158)	0.113 (64/56428)	0.152 (298)	–
MCI22	42	C	MA	8921	G > A	−/+	*MT-ATP6*	G132D	VUS+	0	H	0.000 (0/600)	0.014 (8/56417)	0.024 (47)	Yes^f^
MCI24	65	C	R	8702	C > T	+/−	*MT-ATP6*	T59I	B	+8.05	H1	0.129 (1/773)	0.039 (22/56433)	0.054 (106)	–
MCI27	67	C	MA	8951	T > C	+/−	*MT-ATP6*	V142A	LB	0	T2	0.000 (0/104)	0.005 (3/56424)	0.019 (38)	Yes^g^
MCI28	62	C	MA	9478	T > C	+/−	*MT-CO3*	V91A	LB	−8.95	U7	0.000 (0/27)	0.225 (127/56427)	0.027 (53)	Yes^h^
MCI14	22	S	HA	942	A > G	+/−	*MT-RNR1*	–	N/A	–	J1b	0.000 (0/464)	0.057 (32/56430)	0.053 (103)	–
MCI21	65	C	R	8623	A > G	+/−	*MT-ATP6*	T33A	B	0	H6a	0.000 (0/410)	0.014 (8/56419)	0.02 (39)	–
MCI37	20	S	MA	8412	T > C	+/−	*MT-ATP8*	M16T	B	−4.35	J1b	0.431 (2/464)	0.046 (26/56428)	0.032 (63)	Yes^i^
				5080	A > G	−/+	*MT-ND2*	N204S	LB	+0.36		0.000 (0/464)	0.011 (6/56432)	0.006 (12)	–
MCI39	1	S	MA	3565	A > G	+/−	*MT-ND1*	T87A	B	+0.62	R5a	0.000 (0/55)	0.129 (73/56419)	0.089 (174)	–

a Cardiac Phenotype: C-coronaropathy, S-structural heart disease.

b Physical activity: R –at rest, MA–moderate activity, HA – high activity.

c Ref.Seq. NC_012920.1.

d Apogee2 (Bianco SD et al., 2023 [26]) was applied for pathogenicity prediction of missense variants; B-benign, LB- likely benign, VUS + -variant of unknown significance with a potential pathogenic impact, N/A not available.

e ΔΔG was computed according to Mishra SK 2022 [28].

f Ganetzky RD et al., 2019 [34].

g Formichi P et al., 2020 [36].

h Mkaouar-Rebai E et al., 2011 [38].

i Starikovskaya E et al., 2019 [37].

Furthermore, when the overall distribution of missense variants is represented according to respiratory chain complexes, both complex I and complex V showed a higher number of variants (6/21, 29%, for each complex), suggesting that they may harbor a variation event more frequently than other complexes (Fig. 2b), encouraging a field of further research in SCD pathogenesis.

#### 3.4.1 Haplogroup-unrelated coding mtDNA polymorphisms (SNPs) occur in SCD

Coding SNVs with allele frequency <1% in sample-specific haplogroup branch were considered as SNPs if found in more than 50% of sequences belonging to a different haplogroup, as inferred from GenBank database when at least 10 sequences are recorded for a given haplogroup.

Upon this definition, 11 SNPs were detected in 8 individuals. Two variants were found in rRNA, 4 in tRNA and 5 in protein-coding genes (Table 2, Supplemental Material 4). None of the tRNA variants affected either the amino acid binding at the 3’ terminus nor the anticodon loop, a lack of strong perturbating effect (Supplemental Material 4). The calculation of ΔΔG by PSP-GNM for all the missense variants showed that only the m.8843T>C/*MT-ATP6* variant may affect the protein folding stability (ΔΔG = −6.50kcal/mol). All changes were homoplasmic and 5 variants were previously published in patients with suspected mitochondriopathy (Table 2). In particular, the m.14582A>G/*MT-ND6* variant, which is considered individually neutral, has been shown to act in a synergistic combination with the m.14258G>A when causing LHON [16], suggesting that SNPs may have an additive effect on mitochondria dysfunction such as in the subject MCI4, who also harbored the m.14053A>G/*MT-ND5* variant, or in the background of a different haplogroup. Of note the variant m.5814T>C/*MT-TC* has been described as homoplasmic in a patient with severe encephalomyopathy, but also in other oligosymptomatic relatives, possibly indicating a poor effect by itself [31]. Moreover, the m.3316G>A/*MT-ND1* variant was described as a secondary change in LHON [32], while the m.14687A>G/*MT-TE* variant was observed at high level of heteroplasmy in the muscle biopsy of a subject suffering from myopathy and displaying mitochondrial respiratory chain defects [33]. Eventually, the m.8843T>C/*MT-ATP6* variant, which we predicted as potentially negatively affecting the stability of the protein, was found in a patient whose symptoms were ultimately attributed to an immunogenetic condition, and its equivalent analysis in yeast strain did not show to impair ATP synthase function, supporting the hypothesis that this variant does not have, at least alone, the potential to be disease-causative [34,35].

Therefore, the data from disease databases and databases collecting information from apparently healthy population suggest that natural variation, when uncommon for haplogroup branch type, may potentially modify the risk of SCD with low penetrance effects in a multifactorial aetiology.

#### 3.4.2 Rare coding mtDNA SNVs, mainly involving ATP synthase genes, are associated with SCD

**Fig. 3:**
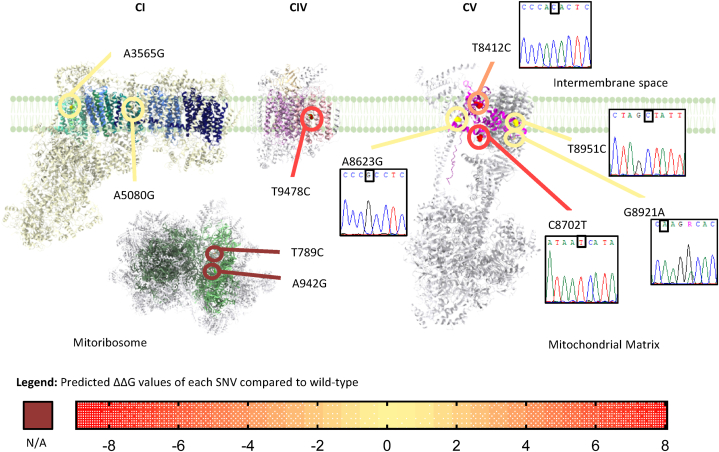
Mitochondrial localization of rare SNVs. For each respiratory chain complex harboring rare SNVs, the mitochondrially–encoded subunits were represented in different shades of color: blue for complex I (CI), pink for complex IV (CIV), violet for complex V (CV), green for mitoribosome. Missense SNVs are indicated by circles colored and displayed according to the relative unfolding Gibbs free energy (ΔΔG) values. A gradient of orange to red shades represents values >+2 and <−2, while a gradient of yellow shades represents scores between −2 and +2. Brown indicates not assessed ΔΔG (N/A). Complex structures were retrieved from PDB (5XTD for complex I, 5Z62 for complex IV, 8H9S for complex V, 3J9M for mitoribosome) and showed by ChimeraX. Chromatograms of *MT-ATP6* variants are displayed.

Rare SNVs were assumed as uncommon SNVs within a specific haplogroup background with allele frequency <1% in population databases. Upon this definition, 10 non-recurrent and rare SNVs were detected in 9 subjects, encompassing 2 rRNA and 8 missense variants (Table 3, Fig. 3). Eight out of ten variants were homoplasmic, while 2 were heteroplasmic with an estimated minimum level of 40%. All missense variants were predicted as benign or likely benign using APOGEE 2, except the heteroplasmic m.8921G>A/*MT-ATP6* variant which, albeit of unknown significance, displayed a potentially damaging impact *in silico*. Moreover, for all missense variants the computed ΔΔG showed potentially damaging impact on the thermodynamic stability of 3 additional mutant proteins (Fig. 3): m.8702C>T/*MT-ATP6* (ΔΔG = +8.05kcal/mol), m.8412T>C/*MT-ATP8* (ΔΔG = −4.35kcal/mol) and m.9478T>C/*MT-CO3* (ΔΔG = −8.95kcal/mol). Four missense variants, 2 of which predicted to affect protein stability and 1 annotated as a possibly damaging VUS by APOGEE 2, had been previously described in patients with suspected mitochondrial disease. In particular, the m.8921G>A/*MT-ATP6* was observed in a patient also harboring an unbalanced chromosomal rearrangement, suggesting a potential damaging combination in the onset of symptoms [34]. The m.8951T>C/MT-ATP6 was detected in a patient with ataxia [36], while the m.8412T>C/*MT-ATP6* was shown in a patient with LHON in combination with a known pathogenic mutation and proposed as possible helper change in the disease onset [37]. Of note, the m.9478T>C/*MT-CO3* variant was identified in heteroplasmy in 2 siblings with Leigh syndrome, but also in other unaffected relatives [38].

Therefore, considering the prediction tool results and disease report, rare mtDNA SNVs, mainly represented by the mitochondrial-encoded ATP synthase genes, seemed good candidates to act as disease susceptibility factors in a favourable context, suggesting a potential contribution in SCD.

**Fig. 4:**
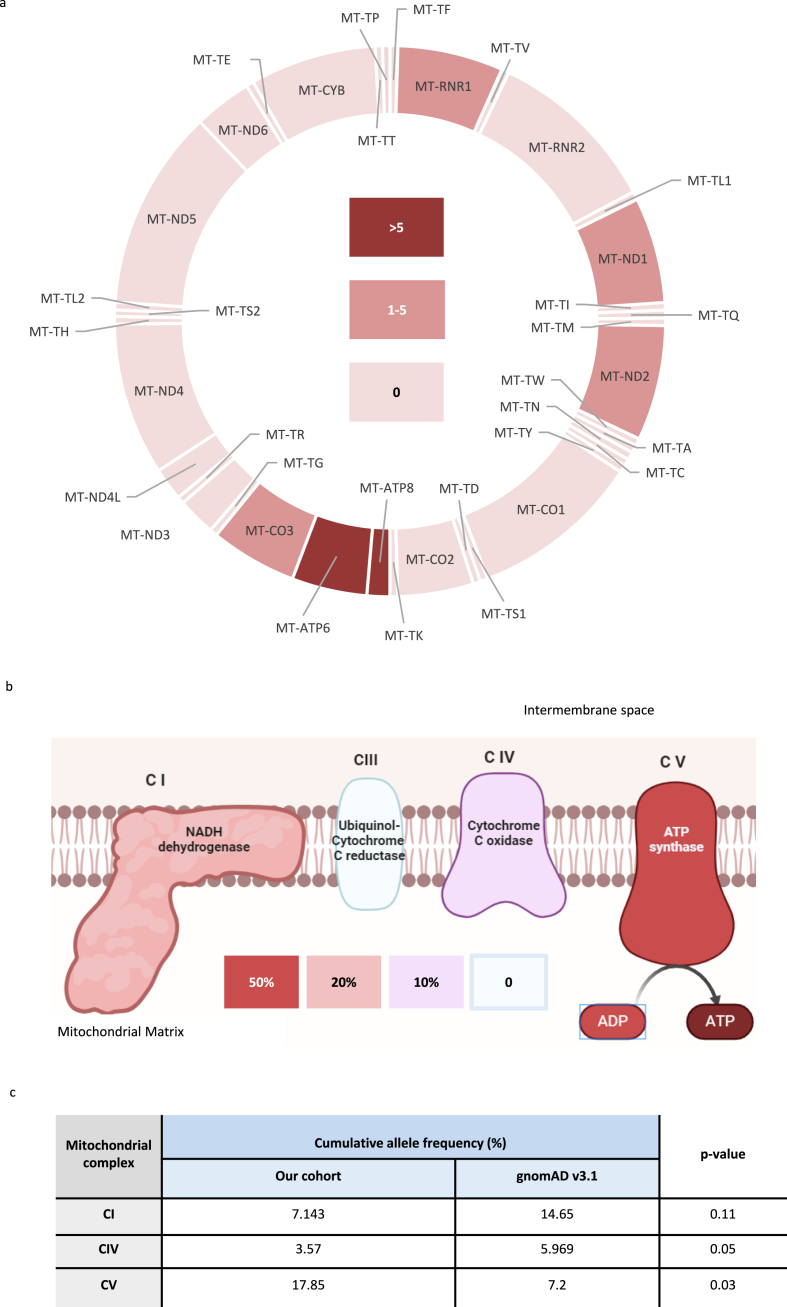
Rare coding mtDNA SNVs, mainly involving ATP synthase genes, are associated with SCD. a) Distribution of rare SNVs identified in 9 patients deceased of SDC according to schematic representation of coding mtDNA gene length. Only rRNA, tRNA and missense SNVs with frequency <1% in public databases were considered. Shades of red indicate the ratio of SNV frequency (counted as single values per gene by the total number of rarer variants) over the gene length percentage in the total mtDNA. b) Distribution of missense SNVs according to the respiratory chain complexes. Only coding SNVs with frequency <1% in public databases were considered. Different colors according to legend depict the mtDNA SNV frequency obtained as single values per complex over the total number of rarer variants. Only complexes with mitochondrial encoded proteins were shown (adapted from Biorender.com). c) Cumulative frequency of rare missense mtDNA variants according to respiratory complex. The allele frequency of rare SNVs in the study cohort was compared to the overall homoplasmic allele frequency of SNVs observed in gnomAD with a rate equal or lower than the highest gnomAD frequency found in our cohort for a rare SNV (0.129%). Using the Chi square test an enrichment in SNV located in complex V was observed in our cohort (p = 0.03). Only mitochondrial complexes harboring rare mtDNA variants in our cohort are shown. CI: complex I, CIV: complex IV, CV: complex V.

As displayed in Fig. 4a, *MT-ATP8* and *MT-ATP6* genes were inferred as hotspots for SNVs with a gene occupancy ratio over 5, meaning that variants occur in these genes at a frequency more than five times higher than expected based on the mtDNA occupancy for that gene.

Consequently to the distribution of rare missense variants according to respiratory chain complexes, complex V showed a cluster of variants (5/10, 50%; Fig. 4b). Therefore, taken together these data might unveil a potential susceptibility role of complex V rare SNVs in SCD that warrants confirmation.

## 4 Discussion

SCD is a global social health issue [3], which in a minority of cases may have a strong genetic origin, being the consequence of cardiac involvement in a primary mitochondriopathy either manifest or latent [39,40]. In this context, mtDNA variants as risk factors in multifactorial heart diseases [8,9] may represent a cause of SCD. Within the mtDNA mutational landscape, we revealed an enrichment of rare haplogroup-unrelated SNVs in complex V (ATP synthase) genes in our pilot. Interestingly, we also observed haplogroup-defining SNVs in subjects belonging to a different haplogroup.

Naturally occurring mtDNA variants marking geographical haplogroup variation have been linked to various disease susceptibility such as LHON or IHD [15,41]. At variance with a previous report of haplogroup H1 adversely affecting non-ischemic SCD risk in a Finnish case-control study [11], in our pilot analysis we did not identify any haplogroup association with SCD. This may be ascribed to our small sample size, but also to different age ranges among cases and controls in the different cohorts, since ethnicity variation, which affects haplogroup frequencies among European countries, may not be ruled out [42].

Variations in oxygen consumption rates and oxidative phosphorylation function occur among different haplogroups [43,44], prompting the study of mtDNA variants in their background context. Interestingly, when we considered the genomes harboring the non haplogroup-defining prioritized coding mtDNA SNVs, the macro-haplogroup distribution mirrored the entire cohort, lacking association with SCD. No difference was found also when the subgroup of rare SNVs located in complex V were considered, where 3/5 subjects belonged to macro-haplogroup H, further strengthening our conclusion that the macro-haplogroup alone may not affect SCD risk.

Since haplogroup backgrounds modulate mitochondrial bioenergetics and penetrance of disease phenotypes [13] naturally occurring mtDNA variation has been called into play in a multifactorial aetiology due to a consolidated role for mitochondrial dysfunction in cardiovascular diseases etiopathogenesis [6,45].

In our cohort, some individuals displayed more than one prioritized variant, including those mapping within mitochondrial tRNAs, which may affect mtDNA-encoded protein synthesis [33]. Further, among prioritized SNPs, some of them were previously reported in suspected mitochondriopathy, in combination with modifier variants [16] or with variable clinical effect [31], which is overall suggestive that these, rather than pathogenic mutations, may underlie a higher SCD risk.

To corroborate our conclusion, among haplogroup-unrelated changes we observed rare mtDNA SNVs mapping in respiratory complex V genes. ATP synthase capitalizes the final steps of mitochondrial oxidative phosphorylation, on which the high energy-consuming cardiac activity strongly relies, whereby ATP synthase dysregulation curbs mitochondrial function and cardiac performance, triggering human heart disease [46]. MtDNA variants in the *MT-ATP6* and *MT-ATP8* genes have been indeed found in patients suffering from congenital heart disease [9,47] as well as other multifactorial cardiovascular disorders [48,49]. Interestingly, although we know that great caution must be applied in comparing small and limited cohorts with larger datasets, when we attempted to contextualize our results, without claims of causal inference, and compared the overall allele frequency of rare SNVs in our cohort to their cumulative frequency in the gnomAD population database, a statistically significant enrichment was detected in our study (10/28–35.7% in our cohort vs 0.65% (0.39% for European non-Finnish population) in gnomAD; p < 0.00001 at Chi-square test). In our opinion, the most striking result in our cohort was a higher frequency than what is expected in the general population of rare ATP synthase variants. Indeed, when the cumulative frequency of observed rare missense variants per mitochondrial complex in our cohort was compared to the cumulative allele frequency of all SNVs detected in gnomAD with a rate equal or lower than the highest gnomAD frequency found in our cohort for a rare SNV (0.129%), an enrichment in complex V variants was observed in our study (5/28–17.86% in our cohort vs 7.2% in gnomAD, p = 0.03 at Chi-square test; Fig. 4c), either in *cis* with other haplogroup-unrelated variant or previously annotated in suspected mitochondriopathies, in combination with modifying factors [34,37]. Intriguingly, such variants were detected in individuals with structural cardiopathy and IHD, 3 of whom displayed a coronary artery disease phenotype and belonged to macro-haplogroup H, a susceptibility factor for IHD [41] in which changes in mitochondrial respiratory chain efficiency have been postulated [43]. Therefore, synergistic factors may lower the threshold for the phenotype exacerbation in a potentially multifactorial way [45].

Despite the limited size of our cohort and the lack of fresh/frozen cardiac tissue for functional analysis, our first attempt sets the basis for the inclusion of mtDNA variation within the risk factors in SCD. It must be noted, indeed, that SNVs in mtDNA non-coding regions, particularly those relevant for replication and transcription, may add up to the list. We here adopted a cautious approach due to the ambiguous interpretation of D-loop variants. In fact, incomplete sequencing/annotation in public databases, the paucity of available functional data and the absence of *in silico* tool predictors, as well as the lack of somatic specimen material needed to ascertain the potential effect of D-loop variants on mtDNA copy number or transcripts, did not prompt us to speculate on their role as risk-associated SNVs.

Altogether, despite the fact that mtDNA-related primary mitochondrial disease may not be a major cause of SCD, at least in our cohort, our results show that haplogroup-unrelated mtDNA SNVs do occur. Regardless of their frequency in population databases, when uncommon for an individual's haplogroup background, we endorse their consideration in a potentially multifactorial aetiology. Finally, the intriguingly high rate of rare ATPase variants raises the hypothesis that low-penetrant dysfunctions in ATP production may affect mitochondrial bioenergetics, potentially modifying the disease risk. Whether such variants may be definitely recognized as risk factors deserves additional investigation, which was here limited by the unavailability of similar matching Italian cohorts. We encourage studies on larger cohorts to corroborate our results in different population settings and strengthen the association between mtDNA variants and SCD risk in a multifactorial interplay.

## CRediT authorship contribution statement

**Elena Luppi:** Writing – original draft, Methodology, Formal analysis, Data curation. **Monica De Luise:** Formal analysis. **Carla Bini:** Investigation, Data curation. **Guido Pelletti:** Investigation, Data curation. **Gaia Tioli:** Formal analysis. **Ivana Kurelac:** Supervision. **Luisa Iommarini:** Supervision, Formal analysis. **Susi Pelotti:** Supervision, Conceptualization. **Giuseppe Gasparre:** Writing – original draft, Supervision, Conceptualization.

## Data availability statement

The data generated during mitochondrial DNA sequencing are available in GenBank database under the accession numbers reported in Supplemental Material 1. All data analyzed during this study are available from the manuscript and the Supplemental information.

## Ethical statement

The study was approved by the Bioethics Committee of the University of Bologna (Prot N. 0131603 May 16, 2023) and performed in compliance with the WMA Declaration of Helsinki.

## Informed consent statement

In light of the nature of the study, informed consent is not necessary as also confirmed by the Bioethics Committee of the University of Bologna (Prot N. 0131603 May 16, 2023)

## Funding

The authors declare that no funds, grants, or other support were received during the preparation of this manuscript.

## Declaration of competing interest

The authors declare that they have no known competing financial interests or personal relationships that could have appeared to influence the work reported in this paper.
