# Supplementary material for: The landscape of rare mitochondrial DNA variants in sudden cardiac death: A potential role for ATP synthase
Source: Heliyon. 2024 Dec 31;11(1):e41592. doi: 10.1016/j.heliyon.2024.e41592 (PMC11759642; doi:10.1016/j.heliyon.2024.e41592)
Supplement: Multimedia component 1 [file mmc1.docx]

**SUPPLEMENTAL MATERIALS**

**Supplemental Material 1**

**Supplemental Material 3**

**Supplemental Material 4**

**The landscape of rare mitochondrial DNA variants in sudden cardiac death: A potential role for ATP synthase**

Elena Luppi1, Monica De Luise1, Carla Bini2, Guido Pelletti2, Gaia Tioli3, Ivana Kurelac1, Luisa Iommarini3, Susi Pelotti2, Giuseppe Gasparre1,*

1. Unit of Medical Genetics, Department of Medical and Surgical Sciences, University of Bologna, Bologna, Italy

2. Unit of Legal Medicine, Department of Medical and Surgical Sciences, University of Bologna, Bologna, Italy

3. Department of Pharmacy and Biotechnology, University of Bologna, Bologna, Italy

***Corresponding Author:**

Giuseppe Gasparre

Department of Medical and Surgical Sciences (DIMEC)

Medical Genetics Unit, Pad. 11

University of Bologna – Medical School

Via Massarenti 9, 40138, Bologna, Italy

Phone: +39 051 2094747

Email: [giuseppe.gasparre3@unibo.it](mailto:giuseppe.gasparre3@unibo.it)

**Supplemental Material 1.** List of study samples and their GenBank ID. For each sample age, sex, cardiac phenotype, activity state before death, nationality and patient-specific haplogroup branch are reported.

| **Patient ID** | | **GenBank ID** | | **Sex and Age (years)** | | **Cardiac Phenotype^a^** | | **Activity state^b^** | **Geographical**  **origin** | | **Patient’s Haplogroup branch** | |
| --- | --- | --- | --- | --- | --- | --- | --- | --- | --- | --- | --- | --- |
| MCI1 | | PP930236 | | M, 41 | | S | | MA | Asian | | M5a | |
| MCI2 | | PP930237 | | M, 36 | | C | | R | European | | H24a | |
| MCI3 | | PP930238 | | M, 45 | | U | | N/A | European | | U1a | |
| MCI4 | | PP930239 | | M, 19 | | S | | N/A | European | | H7 | |
| MCI5 | | PP930240 | | M, 23 | | U | | N/A | European | | U5b | |
| MCI7 | | PP930241 | | M, 24 | | S | | R | European | | N1b | |
| MCI8 | | PP930242 | | M, 16 | | S | | HA | Asian | | U7a | |
| MCI9 | | PP930243 | | M, 56 | | U | | MA | European | | J2a | |
| MCI11 | | PP930244 | | M, 51 | | U | | N/A | European | | H51 | |
| MCI14 | | PP930245 | | M, 22 | | S | | HA | Latin American | | J1b | |
| MCI16 | | PP930246 | | F, 31 | | U | | R | European | | I1b | |
| MCI17 | | PP930247 | | F, 42 | | U | | N/A | European | | H5b | |
| MCI18 | | PP930248 | | M, 65 | | S | | MA | European | | H55b | |
| MCI19 | | PP930249 | | M, 33 | | C | | MA | European | | J2b | |
| **Patient ID** | **GenBank ID** | | **Sex and Age (years)** | | **Cardiac Phenotype^a^** | | **Activity state^b^** | | | **Geographical**  **origin** | | **Patient’s Haplogroup branch** |
| MCI21 | PP930250 | | F, 65 | | C | | R | | | European | | H6a |
| MCI22 | PP930251 | | M, 42 | | C | | MA | | | European | | H |
| MCI23 | PP930252 | | M, 55 | | S | | MA | | | European | | I5a |
| MCI24 | PP930253 | | M, 65 | | C | | R | | | European | | H1 |
| MCI26 | PP930254 | | M, 65 | | C | | MA | | | European | | T2b |
| MCI27 | PP930255 | | M, 67 | | C | | MA | | | European | | T2 |
| MCI28 | PP930256 | | M, 62 | | C | | MA | | | Asian | | U7 |
| MCI29 | PP930257 | | M, 9 | | S | | HA | | | European | | H3a |
| MCI34 | PP930258 | | F, 58 | | U | | R | | | European | | K2a |
| MCI35 | PP930259 | | M, 76 | | C | | MA | | | European | | X2b |
| MCI36 | PP930260 | | M, 66 | | S | | R | | | European | | T2c |
| MCI37 | PP930261 | | M, 20 | | S | | MA | | | European | | J1b |
| MCI38 | PP930262 | | F, 50 | | S | | MA | | | European | | H1b |
| MCI39 | PP930263 | | M, 1 | | S | | MA | | | European | | R5a |

^a^Cardiac Phenotype: C-coronaropathy, S-structural heart disease, U-unspecified heart disease/sudden cardiac death

^b^Physical activity: R –at rest, MA-moderate activity, HA – high activity, N/A - not available

| **Patient ID** | **Age (years)** | **Phenotype ^a^** | **State**  **^b^** | **rCRS Position** | **NT variant** | **Locus** | **Patient’s Haplogroup branch** | **GenBank SeqCount (Frequency) ^c^** | **GenBank frequency % by haplogroup branch (count/total)** | **gnomAD 3.1 Frequency % (count/total)** | **HelixFrequency % in 195983 seqs (count)** |
| --- | --- | --- | --- | --- | --- | --- | --- | --- | --- | --- | --- |
| MCI1 | 41 | S | MA | 16234 | C>T | HVS1/HV1 CR:7S-like | M5a | FL:1736 (2.866%) CR:4367 (5.439%) | 0.000 (0/159) | 1.696% (955/56302) | 1.661% (3255) |
| MCI2 | 36 | C | R | 16180 | A>G | HVS1/HV1CR:7S-like | H24a | FL:30 (0.050%) CR:77 (0.096%) | 0.000 (0/54) | 0.055% (31/56418) | 0.074% (146) |
| MCI3 | 45 | U | N/A | 16148 | C>T | HVS1/HV1 CR:7S-like | U1a | FL:1767 (2.917%) CR:1935 (2.410%) | 0.000 (0/163) | 1.842% (1039/56401) | 0.779% (1527) |
| MCI4 | 19 | S | N/A | 16154 | T>C | HVS1/HV1 CR:7S-like | H7 | FL:79 (0.130%) CR:237 (0.295%) | 0.000 (0/80) | 0.172% (97/56411) | 0.145% (285) |
| MCI5 | 23 | U | N/A | 16256 | C>T | HVS1/HV1 CR:7S-like | U5b | FL:1958 (3.233%) CR:1869 (2.328%) | 0.157 (2/1272) | 4.475% (2521/56338) | 5.27% (10329) |
| MCI11 | 51 | U | N/A | 512 | A>G | HVS3 | H51 | FL:24 (0.040%) CR:26 (0.032%) | 0.000 (0/9) | 0.014% (8/56430) | 0.018% (36) |
| MCI26 | 65 | C | MA | 16270 | C>T | HVS1/HV1 CR:7S-like | T2b | FL:3002 (4.956%) CR:2490 (3.101%) | 0.000 (0/1053) | 8.836% (4980/56361) | 7.629% (14952) |
| **MCI28** | **62** | **C** | **MA** | **185** | **G>A** | **HVS2 CR:OH CR:7S-like** | **U7** | **FL:2424 (4.002%) CR:1586 (1.975%)** | **0.000 (0/27)** | **5.010% (2827/56422)** | **5.392% (10568)** |
| MCI34 | 58 | U | R | 16290 | C>A | HVS1/HV1 CR:7S-like | K2a | FL:7 (0.012%) CR:12 (0.015%) | 0.000 (0/265) | 0.028% (16/56434) | 0.004% (8) |
| MCI35 | 76 | C | MA | 16051 | A>G | HVS1/HV1 | X2b | FL:1557 (2.571%) CR:2003 (2.495%) | 0.369 (1/271) | 2.529% (1427/56416) | 2.58% (5056) |
| MCI38 | 50 | S | MA | 16294 | C>T | HVS1/HV1 CR:7S-like | H1b | FL:5473 (9.036%)CR:5712 (7.114%) | 0.487 (3/616) | 14.190% (7997/56356) | 10.619% (20811) |
| **MCI39** | **1** | **S** | **MA** | **385** | **A>G** | **OH CR:mt3H** | **R5a** | **FL:223 (0.365%) CR:219 (0.270%)** | **0.000 (0/55)** | **0.461% (260/56385)** | **0.375% (734)** |
| MCI14 | 22 | S | HA | 16316 | A>G | HVS1/HV1 CR:7S-like | J1b | FL:465 (0.768% ) CR:1922 (2.394%) | 0.000 (0/464) | 0.618% (349/56431) | 0.607% (1189) |
| **MCI18** | **65** | **S** | **MA** | 71 | G>A | HVS2 CR:7S-like | H55b | FL:6 (0.010%) CR:8 (0.010%) | 0.000 (0/11) | 0.014% (8/56434) | 0.021% (42) |
|  |  |  |  | **207** | **G>A** | **HVS2 CR:OH** |  | **FL:2803 (4.628%) CR:2073 (2.582%)** | **0.000 (0/11)** | **3.649% (2058/56403)** | **3.789% (7426)** |
|  |  |  |  | **373** | **A>G** | **OH CR:mt4H** |  | **FL:207 (0.342%) CR:117 (0.146%)** | **0.000 (0/11)** | **0.085% (48/56425)** | **0.097% (191)** |
|  |  |  |  | 16292 | C>T | HVS1/HV1CR:7S-like |  | FL:1531 (2.528%) CR:1862 (2.319%) | 0.000 (0/11) | 3.466% (1954/56371) | 2.477% (4854) |
| **MCI29** | **9** | **S** | **HA** | **316** | **G>A** | **HVS2CR:OH** | **H3a** | **FL:794 (1.311%) CR:650 (0.810%)** | **0.000 (0/196)** | **2.830% (1595/56366)** | **-** |
| MCI36 | 66 | S | R | 16153 | G>A | HVS1/HV1CR:7S-like | T2c | FL:457 (0.754%  CR:530 (0.660%) | 0.000 (0/286) | 0.834% (470/56340) | 0.837%(1641) |
| MCI37 | 20 | S | MA | 16497 | A>G | 7S-like | J1b | FL:238 (0.393%) CR:355 (0.442%) | 0.216 (1/464) | 0.259% (146/56388) | 0.24% (470) |

**Supplemental Material 3.** mtDNA DLOOP SNVs with frequency <1% in patient’s haplogroup branch. All SNVs were detected in homoplasmy. Entries in bold refer to the H-strand origin (OH) according to mtDNA functional locations on MITOMAP. CR control region sequences, FL full lenght sequences, HVS1/HV1 Hypervariable segment 1, HVS2 Hypervariable segment 2, HVS3 Hypervariable segment 3, mt3H mt3 H-strand control element, mt4H mt4 H-strand control element, NT nucleotide, 7S-like 7S DNA.

^a^Cardiac Phenotype: C-coronaropathy, S-structural heart disease, U-unspecified heart disease/sudden cardiac death

^b^ Physical activity: R – at rest, MA - moderate activity, HA – high activity , N/A - not available ^c^FL: full lenght sequences; CR: control region sequences

**Supplemental Material 4.** DNA sequences (NC_01290.1) of human mitochondrial tRNA genes *MT-TC, MT-TE, MT-TS2, MT-TT*. Predicted secondary structural domains are marked in different colors and adapted from MINTbase v2.0 (Pliatsika V et al. 2018 https://jdc.jefferson.edu/tjucompmedctrfp/23). Variant of interest are circled in dark red.

**Legend:**

**3’ discriminator base**

**D loop**

**Anticodon loop**

**Anticodon**

**T loop**

***MT-TC***

AGCTCCGAGGTGATTTTCATATTGAATTGCAAATTCGAAGAAGCAGCTTCAAACCTGCCGGGGCTT

***MT-TE***

GTTCTTGTAGTTGAAATACAACGATGGTTTTTCATATCATTGGTCGTGGTTGTAGTCCGTGCGAGAATA

***MT-TS2***

GAGAAAGCTCACAAGAACTGCTAACTCATGCCCCCATGTCTAACAACATGGCTTTCTCA

***MT-TT***

GTCCTTGTAGTATAAACTAATACACCAGTCTTGTAAACCGGAGATGAAAACCTTTTTCCAAGGACA
